# Nitrogen fertilization affects maize grain yield through regulating nitrogen uptake, radiation and water use efficiency, photosynthesis and root distribution

**DOI:** 10.7717/peerj.10291

**Published:** 2020-11-16

**Authors:** Wennan Su, Shakeel Ahmad, Irshad Ahmad, Qingfang Han

**Affiliations:** 1 Key Laboratory of Agricultural Soil and Water Engineering in Arid and Semi-arid Areas, Ministry of Education/Institute of Water Saving Agriculture in Arid Areas of China, Northwest Agriculture and Forestry University, Yangling, China; 2 Key Laboratory of Crop Physio-ecology and Tillage Science in North-western Loess Plateau, Ministry of Agriculture/College of Agronomy, Northwest Agriculture and Forestry University, Yangling, China; 3 College of Agronomy, Northwest Agriculture and Forestry University, Yangling, China

**Keywords:** Maize, Nitrogen reduction, Root system, Resource use efficiency, Photosynthesis characteristics

## Abstract

High external nitrogen (N) inputs can maximize maize yield but can cause a subsequent reduction in N use efficiency (NUE). Thus, it is necessary to identify the minimum effective N fertilizer input that does not affect maize grain yield (GY) and to investigate the photosynthetic and root system consequences of this optimal dose. We conducted a 4-year field experiment from 2014 to 2017 with four N application rates: 300 (N_300_), 225 (N_225_), 150 (N_150_), and 0 Kg ha^−1^ (N_0_) in the Northwest of China. GY was assessed by measuring the photosynthetic capacity and root system (root volume, surface area, length density and distribution). Grain yield decreased by −3%, 7.7%, and 21.9% when the N application rates decreased by 25%, 50%, and 100% from 300 Kg ha^−1^. We found that yield reduction driven by N reduction was primarily due to decreased radiation use efficiency (RUE) and WUE instead of intercepted photosynthetically active radiation and evapotranspiration. In the N_225_ treatment, GY, WUE, and RUE were not significantly reduced, or in some cases, were greater than those of the N_300_ treatment. This pattern was also observed with relevant photosynthetic and root attributes (i.e., high net photosynthetic rate, stomatal conductance, and root weight, as well as deep root distribution). Our results suggest that application of N at 225 Kg ha^−1^ can increased yield by improving the RUE, WUE, and NUE in semi-arid regions.

## Introduction

In the past four decades, global maize production has greatly increased (FAO, 2018) mainly due to application of nitrogen (N) fertilizers. Worldwide, N fertilizer has widely been excessively applied to achieve higher grain yields (Cui et al., 2009; Meng et al., 2016; Liang et al., 2020). For example, the average dose of N fertilizer applied by the farmers was greater than 300 Kg ha^−1^ (288 ± 113 Kg ha^−1^), which far exceeds the optimal N rates for maize demonstrated in field experiments (Zhang et al., 2015a; Chang et al., 2014; Yang et al., 2017). N fertilizer was applied in excess (350−600 Kg ha^−1^ year^−1^, Mueller et al., 2013) in an attempt to maximize yields in the North China Plain. However, excessive application of N fertilizer has negative effects on crops, greatly reduces N use efficiency (NUE), and causes significant nitrate leaching losses (more than 50% N to the environment) and contamination of groundwater (Erisman et al., 2013; Wang et al., 2014, 2019a; McBratney & Field, 2015; Ahmad et al., 2018; Suchy et al., 2018). Reducing N input rates from this level to “moderate” levels in maize fields may improve NUE, maintain a fair level of maize grain yield (Zhao et al., 2010; Dai et al., 2015, Qiang et al., 2019), and display less negative environmental impacts. In order to implement reduced N input rates, it is necessary to assess the extent to which the N fertilizer application rate is consistent with crop N requirements to maximize resource utilization and maintain relatively high grain yields (Robertson & Vitousek, 2009; Zhang et al., 2015b).

Radiation interception and radiation use efficiency (RUE) form the basic framework for analyzing crop yield constraints. Variations in crop biomass due to abiotic factors may be attributed to intercepted photosynthetically active radiation (IPAR), RUE, or the combination of both IPAR and RUE (Fletcher et al., 2013). Reduced leaf area under low-N conditions is accompanied by a reduction in radiation interception (Massignam et al., 2012). Low-N conditions primarily decrease the photosynthetic rate per unit area (Vos, Van Der Putten & Birch, 2005), indicating that a low N effect on both leaf size and photosynthetic capacity may affect the final grain yield. Understanding how maize production and resource use are affected by varying N application rates will inform improvement of N fertilizer management to achieve optimal grain yield and resource use efficiency. The appropriate amount of N fertilizer input can improve utilization of precipitation during the crop season (Dai et al., 2015; Herrera et al., 2016; Yang et al., 2017; Qiang et al., 2019). Previous studies of the effect of N fertigation on grain yield and resource use efficiency primarily describe the effects of single resource utilization at the leaf or plant level (Brown, Jamieson & Moot, 2012). A preceding study described the relationship between maize water use efficiency (WUE) and NUE in pot conditions (Wang et al., 2019b). However, few comprehensive studies on the effects of N fertilizer on the utilization of radiation, water, and N resources in field crops have been performed.

Enhancing photosynthesis is critical to promoting crop yield (Mu et al., 2017). In order to understand the underlying mechanisms and differences in photosynthetic capacity, it is necessary to determine the relevant photosynthetic parameters of ear leaves (Li et al., 2013; Zhang et al., 2017; Lamptey et al., 2017). Root morphology and distribution also play key roles in the acquisition of soil resources such as nutrients and water (Ristova & Busch, 2014; Lynch, 2013; Yu et al., 2014). Mi et al. (2010) proposed the ideotype root architecture of “high yield and high N efficiency” in maize, which provided references for root research. In field conditions, the complexity of root sampling has limited efforts to understand the effects of N on roots to shoots. Such research has been conducted under pot conditions (Wang et al., 2019b); thus, it is not possible to assess variation due to differences in light and temperature conditions as well as nitrate N leaching, as occurs in field conditions. Limited knowledge about shoot and root traits related to maize grain yield, NUE, RUE, and WUE under field conditions was investigated. Therefore, exploring the response of photosynthetic parameters and root development to grain yield reduction due to N reduction will provide an important reference for management of N fertilizer inputs.

In the current study, we determined that N reduction results in maintenance of photosynthetic activity and root development to maximize grain yield and radiation, water, and N use by maize crops. The objectives of this study were to (1) investigate the effects of N reduction on water, radiation, and N use efficiencies in maize crops, and (2) determine the effects of N reduction on photosynthetic activity and root development in maize crops under semi-arid conditions. Our findings provide key data to support enhanced maize production and resource use efficiency.

## Materials and Methods

### Field experiments

**Figure 1:**
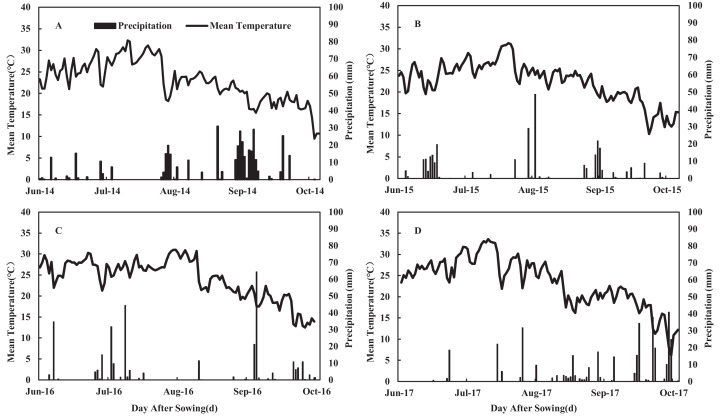
Daily mean temperature and precipitation during the maize growing seasons in 2014 (A), 2015 (B), 2016 (C) and 2017 (D) at the experimental site.

A 4-year field experiment was conducted on the same land at the agricultural experimental station of Northwest A&F University in Yangling (34°20′N, 108°04′E, elevation, 455 m), Shaanxi province, China in 2014, 2015, 2016, and 2017. The experimental site experienced an annual average daylight of 2,150 h, an annual average temperature of 12–14 °C, and an average annual precipitation of 581 mm. The annual mean temperature and total precipitation of the experimental area are shown in Fig. 1. The soil of the experimental site is classified as a dark loess soil, and the former crop was winter wheat. Before sowing, soil chemical properties were analyzed in the top 60 cm of soil for organic matter content, N, phosphorus (P), and potassium (K) (Table 1).

**Table 1:** Soil chemical properties of the top 0–60 cm layers in the experimental fields.

Layer (cm)	Organic matter (g Kg^−1^)	Total nitrogen (g Kg^−1^)	Alkaline nitrogen (mg Kg^−1^)	Available phosphorus (mg Kg^−1^)	Available potassium (mg Kg^−1^)
0–20	15.43	1.203	67.38	12.19	161.17
20–40	14.02	0.987	44.89	9.03	117.21
40–60	13.46	1.069	45.98	6.96	102.67

The research work was carried out with a randomized complete block design with three replications. The total sub plot size was 39 m^2^ (7.8 m long and 5 m wide). The row-to-row spacing was maintained at 60 cm and plant-to-plant spacing at 25 cm. The maize seeds were planted manually, and in each hill, two seeds were sown at a depth of 5 cm in the middle of June during each growing season. Plants were thinned manually for normal plant population densities in the area of (67,500 pl. ha) at V3 (three-leaf stage) (Ritchie & Hanway, 1982). Plots were kept free of weeds, insects, and diseases; and the weeding was controlled by hand and hoe during each growing season. The N treatments applied in this study were: (1) no N (N_0_, 100% reduction from N_300_); (2) 150 Kg N ha^−1^ (N_150_, 50% reduction from N_300_); (3) 225 Kg N ha^−1^ (N_225_, 25% reduction from N_300_); and (4) 300 Kg N ha^−1^ (N_300,_ the traditional N dose applied by farmers in the Loess Plateau of China). Fertilizer N was sourced from urea (46% N), evenly split in the fractions of 1/2 at pre-sowing and side-banded deep (5 cm) into the soil on the sowing rows of 1/2 at the twelve-leaf stage. A total of 150 Kg of phosphorus (calcium superphosphate, P_2_O_5_ 16%) ha^−1^ and 150 Kg of potassium (as potassium sulfate, K_2_O 45%) ha^−1^ were applied 5 days before sowing. Irrigation was applied at the twelve-leaf stage (75 mm). The amount of irrigation was controlled by a water meter (Zhejiang Ningbo Water Meter Co., Ltd., Ningbo, China).

### Sampling and measurements

#### Intercepted photosynthetically active radiation

During the V6–R3, IPAR was measured every 5–7 days (on clear and sunny days between 11:00 AM and 2:00 PM) using a SunScan Canopy Analyzer (Delta, Cambridge, UK). In each plot, three points were chosen. When measuring, the line sensor was placed horizontally between the two ridges (five cm from the soil surface) and was used to collect three consecutive readings of transmitted photosynthetically active radiation (Chen et al., 2016b). Round-trip observations were used to minimize error.

#### Soil water content

At sowing and maturity, soil water content from the 0 to 200 cm layer was determined using a hand-held soil iron drill (Zhang et al., 2019). Between 0 and 20 cm, samples were collected every 10 cm, and between 20 and 200 cm, samples were collected every 20 cm. Soil samples were stored in a closed aluminum box and weighed before drying, oven-dried at 105 °C for 24 h, and weighed separately. The soil water content of each plot was calculated from the average of three random soil core samples. The soil water content was calculated as the difference between the fresh soil weight minus the dry soil weight divided by the dry soil weight.

#### Photosynthetic parameters and leaf area index

In 2016 and 2017, 20 plants were marked prior to the eight-leaf stage (V8). At V8, the tasseling stage (VT), milking stage (R3), and physiological maturity (R6), the three marked plants per plot were selected to measure the net photosynthetic rate (Pn), intercellular CO_2_ concentration (Ci), and stomatal conductance (Gs) on the ear leaves (at VT, R3, and R6) or fully expanded leaves at the top of a plant (at V8) using a photosynthesis analyzer system (LI-6400, LI-COR, Lincoln, NE, USA) on a clear sunny day between 9:00 AM and 11:00 AM (Zhang et al., 2017). The same plants were used for measuring the green leaf area at V8, VT, R3, and R6. The green leaf area index (LAI) was calculated as follows (Birch, Vos & Van Der Putten, 2003):

LAI = 0.75 × Leaf length × maximum width × number of plants within a unit area of land/area of land.

### Root system

Three plants root were sampled using the soil profile method (Holanda et al., 1998) at the V8, VT, R3, and R6 stages of maize. Each root system was excavated from an area of 0.15 m^2^ (line spacing 0.6 m × row spacing 0.25 m) soil around the center of the plant. Root sampling was conducted at depth intervals of 0–30 cm (surface soil layer), 30–60 cm (middle soil layer), and 60–90 cm (deep soil layer) in each plot. Excavated roots were immersed overnight in a plastic container filled with water and washed with tap water on a 0.25-mm screen until the roots were free of soil. Roots were scanned using an HP Scanjet 8200 scanner, and each root image was analyzed using a root analysis program (WinRhizo Provision 5.0; Regent Instruments Inc., Sainte-Foy, QC, Canada) to obtain the root surface area (cm^2^ plant^−1^) and root length (mm). Root volume was measured by the drainage method. Root length density was calculated as the average of three plants’ root lengths divided by the soil volume (Li et al., 2010). Root samples were dried for 48h at 70 °C in an oven to obtain the root dry weight per plant.

### Biomass yield, shoot n content, and grain yield

Four central rows were harvested randomly at 20 m^2^ to measure the grain yield at harvest in each plot. Ten ears were randomly selected from each plot and threshed separately to determine moisture content and kernel number. Grain yield was estimated based on kernel weight and water content and expressed as 14% (w/w) moisture content. Six plants were sampled at each plot and were divided into leaves, stems, and grains. Before determining the N concentration, all plant parts were dried at 70 °C for 48 h and weighed. After weighing, the samples were ground into powder using a Wiley-type mill (<1 mm mesh), weighed (0.3–0.4 g), and were mineralized using H_2_SO_4_–H_2_O_2_; then, total N concentration was obtained by using an automatic Kjeldhal microdistillation analyzer (FOSS, Västra Götaland, Sweden, Nelson & Sommers, 1973).

### Statistical analysis

Daily intercepted solar radiation was calculated using the following equation (Liu et al., 2014):

(1) $${\rm LT} = \displaystyle{{\rm PA{R_L}} \over {\rm PA{R_T}}}$$

where LT is the light transmission ratio, PAR_L_ is the IPAR at the bottom of the canopy, and PAR_T_ is the IPAR at the top of the canopy.

The measured IPAR value of the bottom layer was analyzed by two-dimensional interpolation over 1 day to obtain the IPAR of the entire canopy. Then, the IPAR rate obtained by interpolation analysis is multiplied by the incident PAR measured on the corresponding date by the meteorological observatory to determine the amount of canopy IPAR.

Radiation use efficiency was calculated using the following equation:

(2) $${\rm RUE = \displaystyle{{\rm Mh} \over {\sum Q}} \times {10^{ - 7}} \times 100\%}$$

where ∑Q (MJ m^–2^) is the accumulated intercepted solar radiation, h (KJ Kg^−1^) is the heat energy released of per Kg of grain yield, and M is the grain yield (Kg ha^−1^).

Water use efficiency was calculated as:

(3) $${\rm WUE\; } = {\rm \; }\displaystyle{{\rm GY} \over {\rm ET}}$$

where GY (Kg ha^−1^) is the grain yield, and ET (mm) is the evapotranspiration, as calculated as by soil water balance equation (Huang et al., 2005).

The following equations were also used:

Internal N efficiency,

(4) $${\rm INE\; } = {\rm \; }\displaystyle{{\rm GY} \over {\rm SNC}}{\rm \; \; \; }$$

Agronomic N use efficiency,

(5) $${\rm ANE\; } = \displaystyle{{\left( {{\rm GY}{_{Ni}} - {\rm GY}{_{N0}}} \right)} \over {{\rm Ni}}}$$

Apparent N recovery efficiency,

(6) $${\rm REN\; }\left( {\rm \% } \right) = \displaystyle{{\left( {{\rm SNC}{_{Ni}} - {\rm SNC}{_{N0}}} \right)} \over {\rm Ni}} \times 100$$

N harvest index,

(7) $${\rm NHI\; }\left( {\rm \% } \right)= \displaystyle{{\rm GNC} \over {\rm SNC}} \times 100$$

where SNC (Kg ha^−1^) is the shoot N content calculated as biomass (Kg ha^−1^) multiplied by shoot N concentration (Kg Kg^−1^), i (N rates, Kg ha^−1^) is N application rates 150, 225, or 300, GY_Ni_ (Kg ha^−1^) is grain yield in the N application plots, GY_N0_ (Kg ha^−1^) is grain yield in the no-N application plots, and Ni is the N application rate. SNC_Ni_ (Kg ha^−1^) is the shoot N content in N application plots, SNC_N0_ (Kg ha^−1^) is the shoot N content in the no-N application plots. GNC is grain N content (Kg ha^−1^).

The experimental data were organized and processed using Microsoft Excel 2013 and are presented with standard error. SPSS18.0 (SPSS Institute Inc., Chicago, IL, USA) statistical analysis software was used for variance analysis. The data was checked for normality (Kolmogorov–Smirnov test) and homogeneity of variance (Bartlett–Box test). The effects of N rates, years, and their interactions on the measured variables were tested using one- and two-way ANOVAs. To identify significant treatments effects, multiple comparisons among different treatments were performed using Duncan’s multiple range test. Differences with *p* < 0.05 were considered statistically significant.

## Results

### Grain yield, biomass yield, and crop resource utilization

Our results revealed that the year and N application rates showed significant effects on grain yield (GY), biomass yield (BY), grain N content (GNC), agronomic N use efficiency (ANE), apparent N recovery efficiency (REN), and N harvest index (NHI) (Table 2). The interaction between N application rate and year had no significant effect on the above parameters. We observed no significant differences in GY, BY, and GNC between N_225_ and N_300_ in all growing seasons. Compared with N_300_, the GY of N_150_ and N_0_ decreased ranging from 4.7% to 13.6% and 19.7% to 22.8%, respectively, while the grain yield of treatment N_225_ increased ranging from 1.5% to 3.7% averaged of 4 years. Treatment N_300_ increased BY ranging from 28% to 34%, compared with N_0_, while N_225_ increased BY ranging from 27% to 32% in the 4 years. The GY difference among years may be due to the rainfall amount and seasonal distribution. Rainfall was 390, 284, 311, and 371 mm in 2014, 2015, 2016, and 2017, respectively (Fig. 1). Among the experimental years, GY was higher in 2016 and 2017 compared to 2014 and 2015. 2017 was a more suitable year for maize growth. Although the rainfall in 2016 was reduced, the distribution was relatively uniform throughout the growth period. The early rainfall ensured the regularity and vegetative growth of maize seedlings. The lower GY in 2015 can be explained by the reduced rainfall. In 2014, the greater rainfall was mainly due to the large amount of rainfall occurring 70 days after sowing. Continuous rainfall from the silking to the flowering stage affected maize pollination, and severe stalk rot disease occurred during the grain filling stage, which caused pro-senescence (Fig. 1), causing lower GY. The optimal rate of N application increased the AEN, REN, and NHI, except for the AEN of N_150_ during the 2016 growing season. The NHI was highest for the N_225_ and N_150_ (2014, 2015, and 2017) or N_225_ (2016) treatments.

**Table 2:** Effects of nitrogen application rates on grain yield (GY), biomass yield (BY), grain nitrogen content (GNC), N harvest index (NHI) and agronomic N use efficiency (AEN), apparent N recovery efficiency (RNE).

Year	Nitrogen rates	GY (t ha^−1^)	BY (Kg ha^−1^)	GNC (Kg ha^−1^)	NHI	AEN (Kg Kg^−1^)	REN (Kg Kg^−1^)
2014	N_300_	10.2a	18.1a	127a	59.4b	7.6b	38.4b
	N_225_	10.5a	17.9a	128a	64.5a	11.5a	44.5a
	N_150_	9.4a	16.4b	105b	64.3a	9.9ab	43.1ab
	N_0_	7.9b	13.6c	61c	62.0ab	–	–
2015	N_300_	10.7a	18.6a	134a	63.1b	8.1b	35.3b
	N_225_	11.1a	18.6a	133a	67.3a	12.5a	41.0a
	N_150_	10.2a	17.3b	111b	66.1a	12.8a	41.3a
	N_0_	8.3b	14.4c	68c	64.7b	–	–
2016	N_300_	11.3a	20.0a	142a	60.5b	8.5b	38.7b
	N_225_	11.4a	19.8a	134a	63.6a	12.1a	41.6ab
	N_150_	9.7b	18.4b	113b	59.0b	6.9b	48.6a
	N_0_	8.7c	15.6c	70c	59.1b	–	–
2017	N_300_	11.0a	20.0a	137a	61.4b	7.2b	37.2b
	N_225_	11.4a	20.0a	138a	65.1a	11.5a	45.0a
	N_150_	10.5a	18.5b	116b	63.2ab	11.1a	47.7a
	N_0_	8.8b	15.6c	70c	62.7b	–	–
Source of variation							
	Nitrogen rates (N)	**	**	**	**	**	**
	Year (Y)	**	**	**	**	**	**
	N × Y	ns	ns	ns	ns	ns	ns

**Note:**

Means followed by different lowercase letters indicate significantly different (*p* < 0.05) within the same column and the same year. N_300_, N_225_, N_150_ and N_0_ represent application of nitrogen at a rate of 300, 225, 150 and 0 Kg ha^−1^. **Significant at the 0.01 probability level. ns, non-significant

Throughout the crop growth cycle, differences in ET among N treatments were significant (*p* < 0.05, Table 3). During growing seasons 2015, 2016, and 2017, we observed that the rate of N application showed no significant effect on IPAR. The IPAR of N_0_ was less than that of N_300_ in the 2014 growing season. SNC was increased by increasing the N application rate. For N_300_, SNC increased ranging from 73% to 92% compared with N_0_, and N_225_ application increased SNC ranging from 68% to 81% compared with the N_0_ treatment in the 4 years. IEN was increased with a low rate N application. Application of N at 225 Kg ha^−1^ increased IEN ranging from 8.5% to 12.3% compared with N_300_, while N_150_ decreased IEN ranging from 9.7% to 20.7% compared with N_300_ in the 4 years. N application at the N_300_ and N_225_ rates were similar, while N_300_ increased RUE ranging from 19% to 26%, whereas N_225_ increased RUE ranging from 23% to 29% compared with N_0_ in the 4 years. Reduced N application was associated with reduced WUE. Our results showed that N_225_ increased grain WUE ranging from 25% to 26% compared to N_0_ in the 4 years. WUE was also significantly increased in the N_300_ treatment but to a lesser extent (19–22%). RUE and NUE exhibited non-linear responses to the N application rate, indicating that the maximum grain yield can occur with N_150_ or N_225_ treatments. The differential grain yield between N_225_ and N_300_ was relatively small.

**Table 3:** Effect of nitrogen application rates on evapotranspiration (ET), the accumulated intercepted solar radiation (IPAR), shoot nitrogen content (SNC), internal N use efficiency (INE), radiation use efficiencies (RUE), and water use efficiency (WUE).

Year	Nitrogen rate	ET (mm)	IPAR (MJ m^−2^)	SNC (Kg ha^−1^)	IEN (Kg Kg^−1^)	RUE	WUE
2014	N_300_	350a	974a	214a	47.8c	1.96a	29.1a
	N_225_	346a	960a	198a	53.1b	2.05a	30.4a
	N_150_	343a	989a	163b	57.6b	1.77b	27.4b
	N_0_	333b	906b	98c	81.6a	1.63c	23.8c
2015	N_300_	336a	1181a	212a	50.5d	1.69a	31.8a
	N_225_	337a	1179a	198a	55.9c	1.75a	32.9a
	N_150_	317ab	1131a	168b	60.8b	1.68a	32.1a
	N_0_	303b	1138a	106c	78.1a	1.35b	27.2b
2016	N_300_	414a	1141a	234a	48.1c	1.84a	27.2a
	N_225_	416a	1124a	212a	54.0b	1.89a	27.5a
	N_150_	399ab	1120a	191b	51.0b	1.62b	24.4b
	N_0_	396b	1102a	118c	74.2a	1.47c	22.0c
2017	N_300_	420a	1134a	223a	49.5d	1.82a	26.3ab
	N_225_	417a	1121a	213a	53.7c	1.91a	27.5a
	N_150_	409ab	1122a	183b	57.5b	1.75b	25.8b
	N_0_	392b	1100a	112c	79.4a	1.50c	22.6c
Source of variation							
	Nitrogen (N)	*	*	**	**	**	**
	Year (Y)	**	**	**	*	*	**
	N × Y	ns	ns	ns	ns	ns	ns

**Note:**

Means followed by different lowercase letters within each column indicate significantly different (*p* < 0.05). N_300_, N_225_, N_150_ and N_0_ represent application of nitrogen at a rate of 300, 225, 150 and 0 Kg ha^−1^. ET, total evapotranspiration (mm); IPAR, the accumulated intercepted solar radiation (MJ m^−2^), SNC, shoot nitrogen content (Kg ha^−1^), IEN, internal N use efficiency (Kg Kg^−1^); RUE, radiation use efficiency; WUE, water use efficiency. *Significant at the 0.05 probability level. **Significant at the 0.01 probability level. ns, non-significant.

### Photosynthetic parameters and LAI

**Figure 2:**
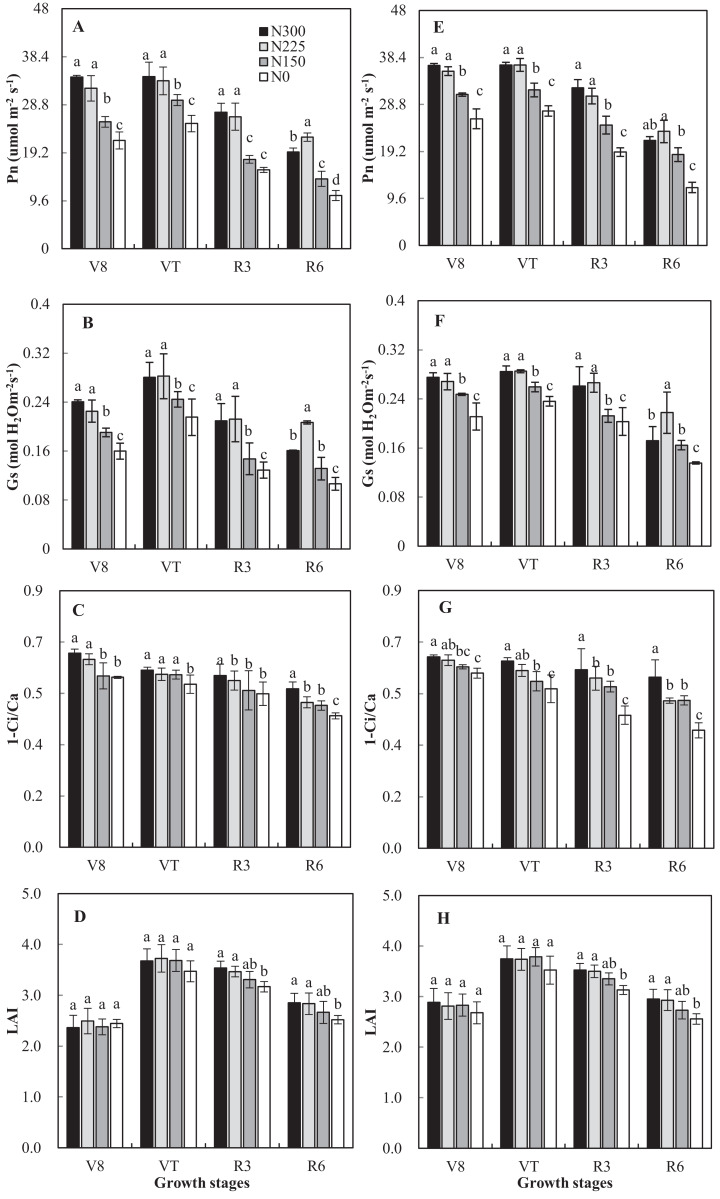
Effects of nitrogen application rates on Pn, Gs, 1-Ci/Ca and leaf area index (LAI) in 2016 (A–D) and 2017 (E–H) growing seasons. N_300_, N_225_, N_150_ and N_0_ represent application of nitrogen at a rate of 300, 225, 150 and 0 Kg ha^−1^. Vertical bars represent the (means ± SD) (*n* = 3).

High external nitrogen application rates and year had significant effects on Pn, Gs, and Ci, but the interaction between N application rates and year did not display significant correlated (Fig. 2). In both 2016 and 2017, the N_0_ and N_150_ treatments resulted in lower values of Pn and Gs when compared with N_225_ and N_300_; however, the effect of N_225_ was relatively greater than that of the N_300_ treatment at R6. This finding indicates that N fertilizer inputs can increase Gs and improve the photosynthetic capacity of maize crops. Conversely, the Pn and Gs of the N_300_ treatment were reduced compared with N_225_. Pn of N_150_, N_225_, and N_300_ increased ranging from 16% to 81% across growth stages compared with N_0_ averaged of 2 years. N application rates significantly affected 1-Ci/Ca (*p* < 0.05). The 1-Ci/Ca of the N_0_ treatment was significantly lower than that of N_300_ in all measurements (Fig. 2).

Overall, the LAI was unaffected (*p* = 0.55) by N application rates, with an average of 2.6 and 3.7 at the V8 and VT stages (Fig. 2). N application rates significantly affected LAI at the R3 and R6 stages. Compared with N_300_, the LAI of N_225_, N_150_ and N_0_ decreased by 1%, 6%, 11%, respectively, and 1%, 7%, and 13%, respectively, at the R3 and R6 stages.

### Root system

The root dry weight reached a maximum value with the N_225_ treatment. Root dry weight of N_225_ increased ranging from 35% to 67% compared with N_0_, while N_300_ increased the root dry weight ranging from 29% to 53% compared with N_0_ average of 2 years (Table 4). We observed clear differences in the vertical distributions of roots as well as root morphology between N application rates, including root volume, root surface area, and root length density, which varied with N application rates and years (Table 5). After the VT stage, the root system distributed in the surface layer (0–30 cm) accounted for greater than 88% of the root weight for all treatments. At the V8 stage, the root ratio was proportional to the N application rate and increased significantly in the middle layer (30–60 cm). In the N_0_ treatment, almost all roots were concentrated in the surface soil layer. The N_0_ treatment exhibited significant increases in the deep-layer (60–90 cm) root ratio at the VT stage. The root ratio in the deep soil layer decreased with increasing N application after the VT stage. At the R3 and R6 stages, the surface root ratio increased with increasing N application rate. For the N_0_ treatment, the deep-layer root ratio decreased after the VT stage. Similar to the dry root weight, root morphology indexes (root volume, surface area, and length density) increased initially and then decreased with increasing N application rate. These indexes reached their maximum values under the N_225_ treatment. At the V8 stage, root morphology indexes were in the order of N_225_ > N_300_ > N_150_ > N_0_ in the surface soil layer, which differed from the middle soil layer (N_300_ > N_225_ > N_150_ > N_0_). Larger roots appeared in deep soil layers at the VT stage when compared to the V8 stage, and the maximum value (root volume, surface area, and length density) for the N_0_ treatment was observed in the middle or deep soil layers. At the R3 and R6 stages, trends in root morphology indexes varied between soil layers; in the surface layer, the indexes followed the order N_225_ > N_300_ > N_150_ > N_0_; in the middle layer, N_225_ > N_150_ > N_300_ > N_0_; and in the deep layer, N_225_ > N_150_ > N_0_ > N_300_.

**Table 4:** Effect of nitrogen application rates on root dry matter and root ratio.

Year	Soil layer	Nitrogen rate	Root dry matter (g plant^−1^)				Root ratio %			
			V8	VT	R3	R6	V8	VT	R3	R6
2016	0–30	N_300_	2.81a	19.89b	19.09ab	16.45b	0.99	0.93	0.93	0.93
		N_225_	3.05a	20.78a	20.43a	17.34a	0.99	0.92	0.92	0.91
		N_150_	1.53b	20.18b	17.50b	14.41b	0.99	0.94	0.92	0.9
		N_0_	0.97c	15.59c	13.19a	10.37c	1	0.89	0.91	0.9
	30–60	N_300_	0.03a	1.52b	1.22b	1.23bc	0.01	0.07	0.06	0.07
		N_225_	0.03a	1.65a	1.45a	1.45a	0.01	0.07	0.07	0.08
		N_150_	0.01b	1.30c	1.26b	1.26b	0.01	0.06	0.07	0.08
		N_0_	0.00b	1.49b	1.22b	0.99c	0	0.08	0.08	0.09
	60–90	N_300_		0.03b	0.13b	0.08c	0	0	0.01	0
		N_225_		0.05b	0.32a	0.34a	0	0	0.01	0.02
		N_150_		0.07b	0.26a	0.26b	0	0	0.01	0.02
		N_0_		0.52a	0.12b	0.12c	0	0.03	0.01	0.01
2017	0–30	N_300_	3.06b	22.09a	19.90ab	17.33b	0.96	0.93	0.93	0.92
		N_225_	4.67a	22.99a	21.90a	18.80a	0.97	0.93	0.92	0.91
		N_150_	2.03c	21.99a	19.42b	15.81b	0.98	0.94	0.91	0.9
		N_0_	1.36c	15.32b	14.20c	11.11c	1	0.88	0.9	0.89
	30–60	N_300_	0.12a	1.52b	1.32c	1.24c	0.04	0.06	0.06	0.07
		N_225_	0.14a	1.65a	1.61a	1.53a	0.03	0.07	0.07	0.07
		N_150_	0.03b	1.30c	1.48b	1.42b	0.02	0.06	0.07	0.08
		N_0_	0.01b	1.49b	1.36c	1.19c	0	0.09	0.09	0.1
	60–90	N_300_		0.03b	0.21c	0.19b	0	0	0.01	0.01
		N_225_		0.05b	0.40a	0.41a	0	0	0.02	0.02
		N_150_		0.07b	0.34b	0.33b	0	0	0.02	0.02
	60–90	N_0_		0.65a	0.21c	0.12b	0	0.04	0.01	0.01

**Note:**

Means followed by different lowercase letters within each column indicate significantly different (*p* < 0.05). N_300_, N_225_, N_150_ and N_0_ represent application of nitrogen at a rate of 300, 225, 150 and 0 Kg ha^−1^.

**Table 5:** Effect of nitrogen application rates on root volume, root surface and root length density.

Year	Soil layer	Nitrogen rate	Root volume (cm^3^ plant^−1^)				Root surface (cm^2^ plant^−1^)				Root length density (mm cm^−3^)			
			V8	VT	R3	R6	V8	VT	R3	R6	V8	VT	R3	R6
2016	0–30	N_300_	31.2a	80.7ab	80.7a	68.6b	306.19a	536.5b	695.8b	569.3b	0.86a	1.24b	0.82b	1.52b
		N_225_	37.7a	91.2a	89.9a	76.6a	339.78a	611.6a	744.9a	639.5a	0.98a	1.54a	2.30a	1.78a
		N_150_	17.3b	86.8b	64.0b	52.4c	167.56b	652.4a	563.5c	472.6c	0.50b	1.23b	1.54c	1.11c
		N_0_	10.7c	54.0c	31.7c	26.9d	132.53c	405.1a	281.8d	228.5d	0.40b	0.90c	0.70d	0.53d
	30–60	N_300_	1.9a	6.7b	6.2a	2.9b	10.53a	101.4b	73.9b	51.1b	0.05a	0.34bc	0.48a	0.18b
		N_225_	0.8b	9.0a	6.7a	5.5a	8.94a	117.4a	138.0a	100.2a	0.03a	0.39b	0.54a	0.4a
		N_150_	0.1c	4.2c	3.3b	2.6b	0.97b	88.5b	90.8b	66.5b	0.003b	0.31c	0.44b	0.35a
		N_0_	0.06c	7.1b	2.9b	1.9c	0.17c	112.5a	14.7c	36.5c	0.000b	0.46a	0.22c	0.13b
	60–90	N_300_		1.1b	2.2b	0.9c		11.1c	44.1c	26.2c		0.01c	0.19c	0.07b
		N_225_		3.2a	5.2a	3.9a		37.4b	118.0a	87.9a		0.17b	0.39a	0.24a
		N_150_		1.5b	3.1b	2.6b		26.1b	91.0b	72.1b		0.07c	0.25b	0.21a
		N_0_		4.6a	2.0b	1.0c		118.0a	47.9c	31.3c		0.35a	0.12c	0.09b
2017	0–30	N_300_	36.8a	92.3b	85.6b	71.6b	428.83b	624.4b	726.8a	611.8b	1.08b	1.37b	1.77b	1.29b
		N_225_	42.9a	112.9a	103.8a	94.3a	520.53a	683.4a	779.8a	690.1a	1.12a	1.74a	3.05a	2.67a
		N_150_	21.2b	100.4a	73.9b	63.5b	248.31c	690.1a	609.6b	487.3c	0.56c	1.43b	1.92b	1.54b
		N_0_	11.8c	58.4c	38.4c	32.6c	166.37d	560.2c	345.4c	286.7d	0.52d	1.00c	0.97c	0.58c
	30–60	N_300_	2.7a	8.3b	7.2b	3.3b	11.37a	115.7a	100.0b	64.2c	0.05a	0.49b	0.57a	0.33b
		N_225_	1.2b	11.0a	8.4a	6.6a	14.15a	133.0b	168.5a	146.1a	0.06a	0.53a	0.60a	0.52a
		N_150_	0.4c	5.5c	4.4bc	3.4b	1.91b	110.4a	110.2b	96.0b	0.02b	0.44b	0.53b	0.48a
		N_0_	0.3c	8.5b	3.8c	2.6b	0.34c	145.5b	29.0c	54.9c	0.03b	0.58a	0.33c	0.25c
	60–90	N_300_		1.4b	3.2b	1.1c		29.3d	76.2b	49.1c		0.09c	0.29c	0.10bc
		N_225_		4.8a	6.3a	4.3a		84.3b	145.7a	126.3a		0.30b	0.47a	0.31a
		N_150_		2.3b	3.7b	3.1b		46.2c	101.1b	87.9b		0.13c	0.36b	0.30b
		N_0_		5.5a	3.0b	2.0c		145.1a	60.2c	36.3c		0.44a	0.20c	0.10c

**Note:**

Means followed by different lowercase letters within each column indicate significantly different (*p* < 0.05). N_300_, N_225_, N_150_ and N_0_ represent application of nitrogen at a rate of 300, 225, 150 and 0 Kg ha^−1^.

## Discussion

An optimal rate of N application is expected to produce a balance between crop demand and N supply and ensure maximum crop production while conserving resources and protecting against environmental damage (Ciampitti & Vyn, 2011; Peng, Li & Fritschi, 2014). Our results showed that during the 2017 growing season, even when photosynthesis was significantly affected by a 50% reduction in N application (N_150_), grain yield was not significantly decreased. In 2016, photosynthetic parameters decreased further for the N_150_ and N_0_ treatments, and the grain yield also decreased significantly. Over 4 years, we observed no significant reduction in grain yield for the N_225_ treatment (Table 2), which even displayed a grain yield higher than that of the N_300_ treatment, although the difference was not significant. Our research indicated that the N dose could be reduced by at least 25% without compromising grain production. Our results are similar to those of Li et al. (2007) and Lamptey et al. (2017), who reported an optimal N fertilization range for summer maize of 200 to 300 Kg N ha^−1^. Under the N_0_ and N_150_ treatments, dry matter accumulation was limited, and the difference between N_300_ and N_225_ treatments was not significant. The reduction in crop yield induced by N reduction can be explained by various factors, as described below.

Our experiments also addressed the mechanism by which maize yield is affected by N application rates. We found that for most years, IPAR was not affected by N fertilizer application rates, a finding similar to that described in previous studies (Vos, Van Der Putten & Birch, 2005; Massignam et al., 2012). N application rate significantly affected RUE (*p* < 0.05). For N_0_ and N_150_, the RUE was significantly less than that of N_300_. However, the RUE of the N_225_-treated crop was greater than that of the N_300_-treated crop. In this case, lower grain yield driven by lower N treatment corresponded to lower RUE. These results indicate that under such production conditions, N application rate mainly affects grain yield by affecting RUE rather than IPAR. Vos, Van Der Putten & Birch (2005) also found that maize tends to sacrifice specific leaf N and RUE while maintaining leaf area (small changes to LAI (Fig. 2) in comparison with the large decrease in Pn (Fig. 2) at low N application rates). Therefore, in the condition of high IPAR, improving the RUE may represent a valid mechanism to achieve high maize grain yield.

The actual ET involves two components: crop transpiration and soil evaporation. N application can increase ET during the reproductive period due to high leaf transpiration under high N conditions (Lamptey et al., 2017; Rudnick et al., 2017). The relatively low sensitivity of IPAR to N supply in maize may also be consistent with the low sensitivity of soil evaporation to N supply. In this case, the lower grain yields associated with the N_0_ treatments corresponded to lower ET and WUE values. In addition, WUE of the N_300_-treated crop was lower than that of the N_225_-treated crop. This is due to a higher sensitivity to soil water by the plant at higher N application rates. An increase in N application rates is usually accompanied by a decrease in NUE (Ju et al., 2015). Significant effects of N were also observed on N use efficiency (NUE including AEN, REN, NHI, and INE) in our study. Conversely, reducing the N application rate can create a balance between crop demand and N supply (Lamptey et al., 2017). The desired N concentration of the plant, under the N_0_ treatment, has a high INE value, indicating that the plant N concentration or yield are low and that the amount of N in the plant absorbed from the soil is small. N accumulation increased with increasing N application, but N accumulation in the grain was not significantly different between N_225_ and N_300_ treatments, and the value of NHI at N_225_ was greater than that at N_300_, which indicated that increased N in the plant did not transfer to the grain, resulting in excessive N absorption and residual N in the vegetative organs.

The plasticity of root morphology allows it to respond to soil mineral nutrients (Peng, Li & Li, 2012; Yu et al., 2014). We found that root dry weight reached a maximum with the N_225_ treatment, which suggested that the relationship between N input and the root system is not linear and positive; N input may even have a negative impact on root growth and development. In the present study, application of N fertilizer promoted growth of roots in the 0–60 cm soil layer and increased the proportion of roots in this layer, indicating that N application improved growth of the upper layer roots. In the late growth stage (after VT), the N_0_ treatment exhibited a negative effect on the root dry weight and proportion in the 30–60 cm and 60–90 cm soil layers, indicating that N deficiency would be detrimental during the accelerated aging of deep layer roots. Not only are root morphology and nutrient absorption closely related, but the spatial distribution of roots is also closely connected to crop growth and productivity (Mi et al., 2010; Lynch, 2013). In both years, the root system exhibited the optimal distribution under the N_225_ treatment, with a higher root length density in the observed soil layer, resulting in larger and deeper infiltration scales. Slower root senescence in the N_225_ treatment is also a major contributor to N rate–induced increases in grain yield. Studies have shown that the relative stability of the deep root environment is beneficial in promoting the buffer capacity of the root system in adverse soil environments and achieving high grain yield and resource use efficiency (Chen et al., 2010; Mi et al., 2010; Wasson et al., 2012; Saengwilai et al., 2014). The results of our study during the 2016 and 2017 growing seasons showed that excessive N (N_300_) application negatively affects early deep root growth compared with N_225_. High external N input also appeared to generate an overall inhibitory effect on later root growth. There are many reasons for the observed reduction in crop yield. Slight reductions in crop yield induced by excessive N application may be due to negative impacts on root growth during the early growth stage or may be caused by differing mechanisms of aging leading to N loss and relative N deficiency during the reproductive stage. N deficiency induces root thinning and increases longitudinal expansion by promoting root growth in the deep soil, while high N inhibits vertical expansion of roots (Trachsel et al., 2013; Mu et al., 2015). This study explained the effect of excessive N and N deficiency on yield from the perspective of root morphology and growth.

The role of N in grain formation is mainly explained by photosynthesis, and N reduction usually negatively impacts photosynthetic performance in maize (Massignam et al., 2012; Olszewski et al., 2014). Although the N_150_ treatment significantly reduced Pn in the 2017 growing season, the grain yield for the N_150_ treatment was not significantly reduced, indicating that the plant’s transient photosynthetic parameters were more sensitive than the dry mass factors in responding to changes in N application rates. Our results illustrate the physiological basis for utilizing the 225 Kg ha^−1^ N rate to improve the stress resistance of summer maize plants in the Loess Plateau. For the N_300_ treatment, decreased Pn was due to lower Gs, which may be due to N-associated increased sensitivity of plant to soil water and results in lower WUE than the N_225_ treatment at the R6 stages. In the low-N treatment, maize plants showed reduced leaf rolling under soil water stress compared with the high N treatment and thus obtained higher WUE (Wang et al., 2019b).

At different measurement dates, the maximum value of Gs was not always associated with the N_300_ treatment (Fig. 2). In general, plants’ water requirements are expected be greater under high N conditions. Thus, high-N fertilizer plots are more likely to be water-deficient if the soil moisture is inadequate, which aggravates the plant’s stomatal limitations (1-Ci/Ca) and resulting in reduction of Gs and Pn. Similar findings have been described in wheat (Zhang et al., 2017). Previous studies also pointed out that under soil water-stress, ABA (as the signal carrier) transmitted to the shoot, reducing the Gs and increasing stomatal limitations in the initial drought (Larcher, 2003; Li & Xu, 2014; Yan et al., 2017). The inadequate soil moisture of the N_300_ treatment is not serious enough to affect non-stomatal factors restricting photosynthetic carbon assimilation. The Pn of the N_225_ treatment was similar to that of the N_300_ treatment during the VT stage, but it was less than that of the N_225_ treatment in R6. Although the N_225_ treatment showed a higher photosynthetic rate in the R6 than the N_300_ treatment, the ratio did not result in a significantly higher yield, which may be caused by the small contribution of high photosynthetic capacity to a grain yield (Acciaresi et al., 2014). In addition, the photosynthetic capacity may also be related to the differences in N nutrition characteristics among different maize genotypes (Chen et al., 2014; Li et al., 2015). Thus, while the application rate of 225 Kg N ha^−1^ was may still be high, the current N rate was relatively effective in improving resource use efficiencies. Under production conditions, large amounts of N input are often used as an “insurance” against higher yields to ensure further increases in maize production (Huang et al., 2007; Chen et al., 2012). However, this behavior results in a significant reduction in NUE (Chen et al., 2010) and a slight reduction of RUE and WUE. Previously, Chen et al. (2016a) and Mu et al. (2018) reported that N is used primarily for cell morphogenesis, that N in leaves is N redundant, and excess N is mainly stored in soluble protein and light-harvesting pigment-protein complexes. Therefore, in our study, the grain yield of N_225_ treatment did not display significantly different results compared to the N_300_ treatment, and the root system and photosynthetic capacity showed certain advantages of N_225_ treatment. From these findings, we can conclude that it is achievable to improve resource use efficiencies while ensuring grain yield. Actually, maize genotypes and soil moisture also affect GY and physiological characteristics (Ciampitti et al., 2013). In the current experiment, these factors are not taken into consideration. In future studies, we will focus on the effects of genotype with the aim of maximizing GY and resource use efficiency.

## Conclusions

Decreased grain yield due to N reduction was driven by reduced radiation utilization efficiency and WUE; the impact of radiation interception and total water evapotranspiration were relatively small. An application rate of 225 Kg N ha^−1^ could be used as a reference for optimal N application in the Loess Plateau of China. This N application rate optimized the eco-physiological responses of the plant, a finding which was confirmed by measuring photosynthetic activity and the root system. This response to optimizing N input resulted in higher grain yield, RUE, WUE, and NUE. Reducing N application rates has strong recoverability in maize production and can maximize the capture and utilization of resources, increasing the maize grain yield.

## Supplemental Information

10.7717/peerj.10291/supp-1 Supplemental Information 1 Raw data.

## Additional Information and Declarations

### Data Availability

The following information was supplied regarding data availability: The raw measurements are available in the Supplemental File.
